# Landscape‐Level Assessment of Topographic Influences on Organic Carbon Storage in Forests of Far Western Nepal

**DOI:** 10.1002/pei3.70039

**Published:** 2025-03-26

**Authors:** Santosh GC, Gandhiv Kafle, Santosh Ayer, Renuka Khamcha, Sandip Poudel, Aman Prabhakar, Amrita Bhusal, Prakash Lamichhane, Janak Airee, Terisa Sapkota

**Affiliations:** ^1^ College of Natural Resource Management (CNRM) Agriculture and Forestry University Katari Nepal; ^2^ Faculty of Forestry Agriculture and Forestry University Hetauda Nepal; ^3^ Faculty of Agricultural, Life & Environmental Sciences University of Alberta Edmonton Alberta Canada; ^4^ Forest Research and Training Centre Kathmandu Nepal

**Keywords:** carbon sequestration, climate change mitigation, forest biomass, topography

## Abstract

Carbon sequestration significantly aids in mitigating climate change, with its spatial distribution greatly influenced by topographical factors. However, data on organic carbon distribution and its interaction with topographic factors inside the forest of the Far Western Region of Nepal are limited. Therefore, this study aims to analyze forest carbon stock variation under different topographic variables (physiographic region, aspect, and slope) in Far‐western Nepal. In this study, stratified systematic cluster sampling was adopted with elevation, aspect, and slope as strata. A total of 181 circular plots were used for dendrometric measurements and soil sample collection. Within each plot, diameter at breast height and height of each tree (diameter at breast height ≥ 5 cm) were measured for biomass carbon assessment. Composite soil samples (0–30 cm) from each soil pit within a plot were collected for determining soil organic carbon stock. Physiographic region‐wise, our study reported the highest mean aboveground carbon (174.04 ± 29.75 ton ha^−1^) and belowground carbon (34.044 ± 5.95 ton ha^−1^) and soil organic carbon stock (150.62 ± 11.02 ton ha^−1^) in the Mountain and High Himal region. The East aspect exhibited the highest aboveground carbon (125.9 ± 22.34 ton ha^−1^) and belowground carbon (27.54 ± 3.44 ton ha^−1^) stocks, while the North aspect showed the highest soil organic carbon stock (96.85 ± 8.82 ton ha^−1^). Organic carbon stocks declined with steeper slopes, with the (0–10)° slope category recording the highest aboveground organic carbon (135.17 ± 17.87 ton ha^−1^), belowground carbon (27.03 ± 3.57 ton ha^−1^), and soil organic carbon (107.14 ± 12.51 ton ha^−1^) stocks. Conversely, the (30–40)° slope category exhibited the lowest organic carbon stocks across all pools. This study's findings will support accurate monitoring, reporting, and verification (MRV) processes for initiatives like reducing emissions from deforestation and forest degradation (REDD+) and enhance credibility on United National Framework Convention on Climate Change (UNFCCC) reporting on a national scale. The design and application of site‐specific management activities to optimize organic carbon storage are recommended due to the observed variability of organic carbon stock with topographic factors.

## 1 Introduction

The worldwide terrestrial carbon (C) cycle depends heavily on forest ecosystems, which actively participate in activities like photosynthesis, carbon storage, and release through the processes of respiration, disintegration, and combustion (Stephenson et al. 2014). Forests, central to the global carbon cycle, store 50%–65% of terrestrial organic carbon, including soil carbon, and account for approximately 80% of all biomass, acting as critical carbon sinks (Reichstein and Carvalhais 2019; Manral et al. 2023). Their ability to sequester a substantial percentage of terrestrial C, ranging from 70% to 90%, is closely tied to plant diversity within various forest types (Pandey et al. 2023). Approximately half of the planet's terrestrial C is held collectively by forests and their associated soils, with forest C representing the largest terrestrial C reserve (Houghton 2007). Nevertheless, these crucial ecosystems are confronting significant challenges due to human‐induced activities, particularly deforestation and changes in land use, resulting in a 30% reduction in C stocks (Awasthi et al. 2022; Manral et al. 2023). With a growing population, expanding agriculture, and infrastructure development, biodiversity faces threats, contributing to elevated CO_2_ levels in the atmosphere (Marques et al. 2019). Beyond their C cycle role, forests provide diverse ecosystem goods and services to local communities (Fartyal et al. 2022). However, the decline in vegetation diversity threatens ecosystem stability, especially given its intrinsic connection to C sequestration (Isbell et al. 2018).

Global research, characterized by extensive surveys of forest biomass and soil, has brought to light widespread geographic trends in C stock distribution. Previous studies such as Nath et al. (2018), Piao et al. (2019), Sheikh et al. (2020), and Yang et al. (2007) underscored the influential role of vegetation types in shaping spatial patterns. Similarly, a comprehensive review of existing studies in Nepal has unveiled significant variations in C stocks among different forest types (Tripathi et al. 2017; DFRS 2014; Ghale et al. 2020; Gurung et al. 2022; Sharma et al. 2020; Ayer et al. 2023; Ayer, Timilsina, et al. 2024). These studies present diverse estimates of C stock, specific to distinct regions and forest categories. For instance, Ghale et al. (2020) and Ayer, Timilsina, et al. (2024) documented C stock in bamboo forests ranging from 113.82 to 148.73 ton ^−1^ha in Western and Eastern Nepal, respectively. In Central Nepal, Gurung et al. (2022) reported mean tree C stock ranging from 22.5 to 87.13 ton ha^−1^ in middle mountain forest types. Similarly, Sharma et al. (2020) reported a mean C stock of 107.5 ton ha^−1^ in *Pinus roxburghii* plantation forest in Kathmandu, Nepal. Additionally, the total biomass and C stock of a community forest dominated by *Schima wallichi* and *Castanopsis indica* were found to be 108.09 and 50.80 ton ha,^−1^ respectively, in Kaski, Nepal (Tripathi et al. 2017). The estimated total C stock of Terai forests and Churia forests of Nepal was reported to be 124.14 ton ha^−1^ and 116.94 ton ha^−1^, respectively (DFRS 2014). These variations in C stocks are caused by a combination of factors such as forest types, the composition of tree species, patterns of land use, and climate condition (Sheikh et al. 2020).

In forest ecosystems, topographical factors such as elevation, aspect, and slope play a crucial role in shaping tree species distribution and influencing C storage dynamics (McEwan et al. 2011; Valencia et al. 2009). Slope and aspect collectively determine local temperature conditions by impacting the amount of sunlight and heat received by different areas of a forest due to their orientation relative to the sun (Holland and Steyn 1975; Bruun et al. 2006). This interaction results in variations in microclimates, where areas with different aspects experience diverse durations and intensities of sunlight (Moeslund et al. 2013). These microclimatic variations are closely tied to C storage, as different aspects affect the amount of solar energy reaching specific forest areas, subsequently influencing plant growth and, consequently, the overall biomass and C stock (Holland and Steyn 1975). Additionally, when considered alongside slope, aspect significantly influences the distribution patterns of vegetation within the forest (Titshall et al. 2000). Slope affects vegetation C by influencing nutrient availability, water availability, root systems, species composition, and forest structure (Sharma et al. 2011; Simegn and Soromessa 2015). Simultaneously, aspect introduces variation in solar radiation and the presence of moisture in the soil (Moeslund et al. 2013). This intricate interplay among elevation, aspect, slope, and other abiotic factors, such as soil conditions and precipitation, underscores the multifaceted nature of C storage in forest ecosystems (Holland and Steyn 1975; Yohannes et al. 2015; Simegn and Soromessa 2015).

While numerous studies have assessed biomass and C stock levels under contrasting management regimes and land use types in Nepal (Joshi et al. 2021; Subedi et al. 2022; Lamsal et al. 2023; Maharjan et al. 2024; Ayer, Timilsina, et al. 2024), a noticeable research gap exists regarding the influence of topographic variables, specifically elevation (physiographic region), slope, and aspect on C dynamics (Bohara et al. 2021; Ayer, Joshi, et al. 2024). In this context, this research was carried out to explore how topographical factors affect C storage in forests in Far‐western Nepal, filling a gap in current understanding. We hypothesized that altitude or physiographic zone, slope, and aspect significantly affect the forest C stock. Based on prior studies and local environmental conditions, we expect higher carbon storage in soil and vegetation in the Mountain and High Himal regions and north‐facing slopes, which typically experience cooler temperatures, higher moisture retention, and reduced decomposition rates. Similarly, steeper slopes may have lower carbon storage because of increased erosion and reduced organic matter accumulation.

## 2 Methodology

### 2.1 Study Area

**FIGURE 1:**
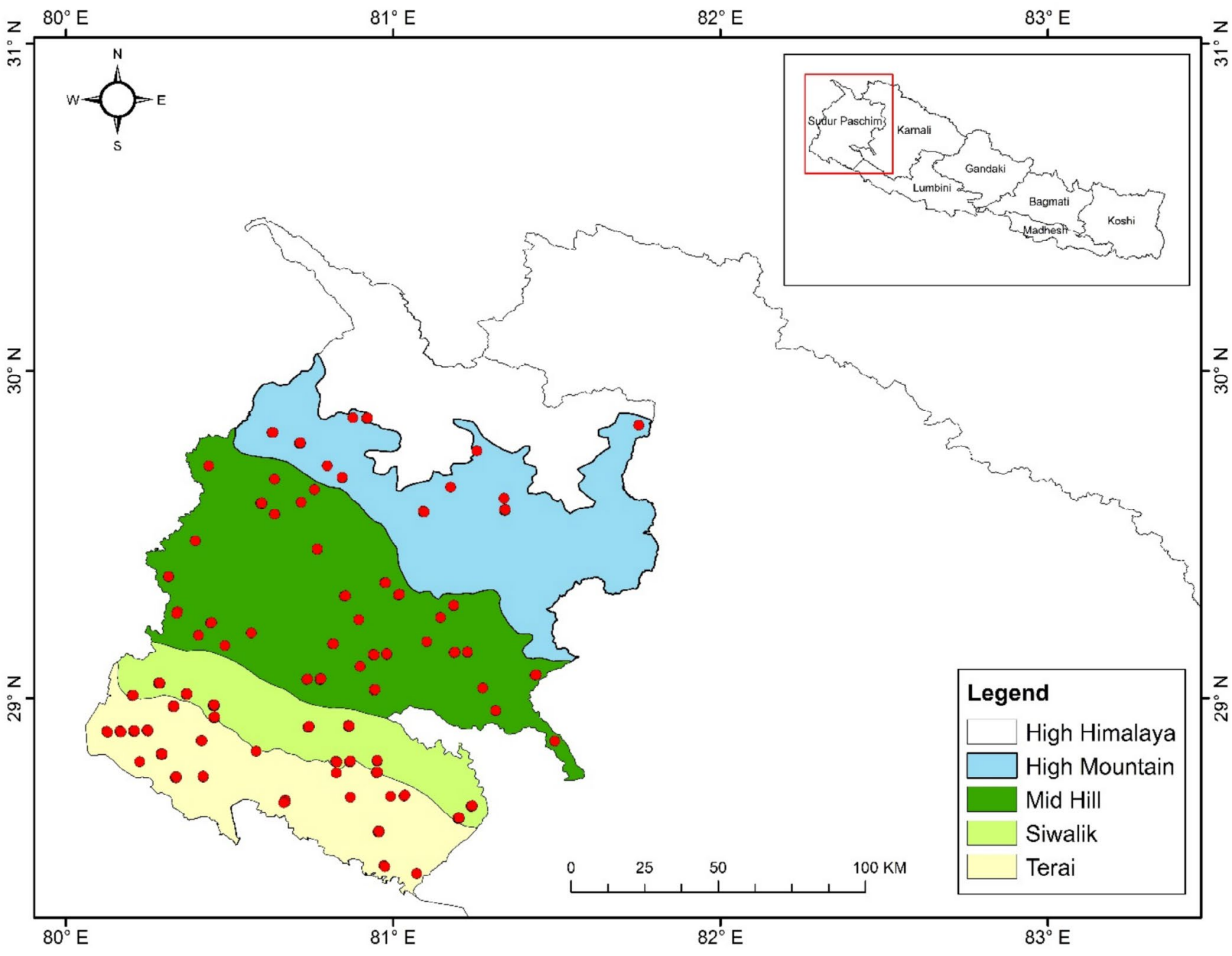
Study area map of Far Western Region showing different physiographic regions: (a) High Himalaya, (b) High mountain, (c) Mid hill, (d) Siwalik and (e) Terai. Red dots represent sample plots for data collection.

This research was conducted within the forested areas of the Far Western Region of Nepal (Easting: 80°34′1″ and Northing: 28°42′12″) which spans 19,539 km^2^ (about 13.22%) of the country's total area (Figure 1). Geographically, the Far Western Region is split into 5 regions: the Terai in southern Nepal, the Siwalik and Mid Hill in the center, and the High Mountains and High Himal in northern Nepal (Upreti 1999). This region comprises 16.94% (1,010,207 ha) of the total Forest of Nepal, out of which Mid Hill (42.05%) has the highest forest area, followed by High Mountains and High Himal (27.48%), Siwalik (17.87%) and Terai region (12.60%) (DFRS 2014). The northern part of Far Western Nepal has chilly summers and harsh winters, whereas the south of the territory has tropical summers and warm winters (Sapkota 2007). Average annual precipitation in Far Western Nepal ranges from 500 to 2500 mm, with the highest average precipitation per annum in the Terai Region (500–2500 mm). A wide range of vegetation types are found in this region, such as grasslands, savannahs and shrublands, ropical and subtropical forests, Temperate Broadleaved forests, Sub‐alpine Conifer forests, and alpine meadows and shrublands (DFRS 2014). The prominent tree species found in the area encompass *Shorea robusta, Dalbergia sissoo, P. roxburghii, S. wallichi, C. indica, Alnus nepalensis, Quercus lamellosa, Abies spectabilis, Betula utilis, Rhododendron species*, and *Juniperus species* (FRTC 2021). The study area was divided into four major aspect categories (East, West, North and South) and five slope categories (0°–10°, 10°–20°, 20°–30°, 30°–40°, and > 40°); however, the > 40° slope category was excluded during the forest inventory due to inaccessibility to those areas (Figure 1).

### 2.2 Sampling Design

As stated by Kleinn (1994), a two‐phase stratified systematic cluster design technique was used for the Forest Resource Assessment (FRA) across Nepal. Initially, the province was stratified into five zones: Terai, Siwalik, Mid Hill, High Mountain, and High Himal based on physiographic regions (Figure 1). A grid of 4 km^2^ was systematically set up in the strata with a cell width of 300 m (DFRS 2014). Using a cluster sampling design, subsample circular plots (20 m radius) were chosen from the sample plots selected in the first step. Two cluster designs were finalized, consisting of six circular plots for four Mountainous regions and four circular plots for the Terai region due to the higher spatial diversity of forest structure and composition in the mountains than in the Terai region (DFRS 2014). A total of 181 sample plots were distributed along two parallel lines, spaced 150 m apart for the mountains and 300 m for the Terai region (DFRS 2014).

### 2.3 Data Collection

Biometric parameters such as tree height and DBH were measured for trees (DBH > 5 cm). Diameter tape and Haglof T3 Transponder Range Finder were used for DBH and height measurement, respectively. Similarly, for soil sample collection, soil pits having a 2 × 2 m area were established in all cardinal directions of the concentric circular sample plots (CCSPs) to collect composite soil samples (up to depth of 30 cm) (DFRS 2014) Figure 2.

**FIGURE 2:**
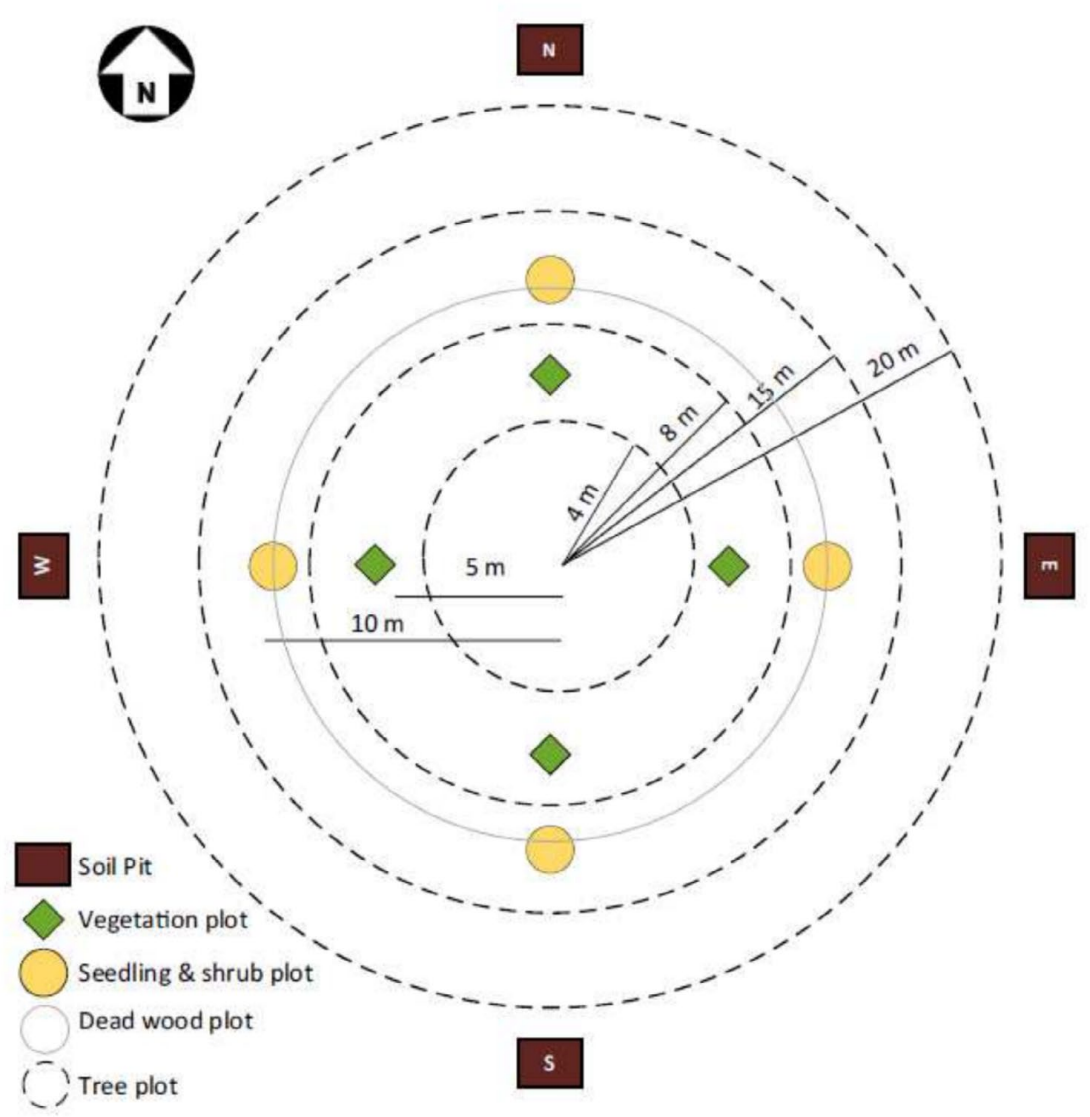
Layout of the CCSPs: (a) Soil pit, (b) Vegetation plot, (c) Seedling and shrub plot, (d) Dead wood plot, and (e) Tree plot. *Source:* DFRS (2014).

The samples were collected by using a 100 mm long, slightly conical cylinder corer with a lower diameter of 37 mm (at its cutting edge) and an upper diameter of 40 mm; the volume of each soil subsample collected was 107.5 cm^3^ (DFRS 2014). In this way a total of four composite soil samples per plot were collected.

### 2.4 Carbon Stock Measurement

#### 2.4.1 Above and Belowground Biomass and Carbon

The volume and biomass of standing trees were calculated using the volume equations developed by Sharma and Pukkala (1990) and the biomass models developed by MoFSC (1988). These equations produced air‐dried biomass values, which were then transformed into oven‐dried biomass values using a conversion factor of 0.91 (Chaturvedi and Singh 1982).

##### 2.4.1.1 Tree Volume Estimation

The following allometric equation developed by Sharma and Pukkala (1990) was used to estimate tree volumes over bark:

(1) $$\ln\left(v\right)=a+b\;\ln\left(d\right)+c\;\ln\left(h\right)$$

where, ln = Natural logarithm to the base 2.71828.

(2) $$v=\text{volume}\;\left(m^{3}\right)=\exp\;\left[a+b\times \ln\;\left(\mathrm{DBH}\right)+c\times \ln\left(h\right)\right]$$

where *d* = DBH (cm), *h* = total tree height (m), *a*, *b*, and *c* are coefficients depending on species.

##### 2.4.1.2 Tree Stem Biomass Estimation

Tree‐stem biomass was calculated using the following formula:

(3) $$\text{Stem biomass}\;\left(\mathrm{Kg}\right)=\mathrm{vol}.\times \text{density}$$

where, vol. = stem volume (m^3^) and density = air‐dried wood density (kg m^−3^).

##### 2.4.1.3 Foliage and Branch Biomass Estimation

Estimating branch and foliage biomass from stem biomass was done using the separate branch‐to‐stem and foliage‐to‐stem biomass ratios outlined by the Ministry of Forest and Soil Conservation MFSC (1988). Then total biomass was calculated using the following formula:

(4) $$\text{Total Biomass}\;\left(\mathrm{Kg}\right)=\text{Stem biomass}+\text{Branch biomass}+\text{Foliage biomass}$$

To estimate below‐ground biomass, (MacDicken 1997) root‐to‐shoot ratio value of 1:5 was used; that is, to estimate below‐ground biomass as 20% of above‐ground tree biomass. The C content in biomass was calculated by multiplying biomass by the default C fraction of 0.47 (Intergovernmental Panel on Climate Change 2006):

(5) $$C\;\text{stock}\;\left(\mathrm{ton}\;{\mathrm{ha}}^{-1}\right)={\text{Biomass}}^{*}\;0.47$$

#### 2.4.2 Soil Organic Carbon (SOC) Stock

SOC concentration was determined in the laboratory following the Walkley‐Black Method (Walkley and Black 1934). Prior to this, soil samples were air‐dried, sieved through a 2 mm mesh, and ground to a fine powder. A known weight of the prepared soil was then oven‐dried at 105°C for 24 h to remove moisture. This soil lab analysis was done at the Forest Research and Training Centre (FRTC) in Kathmandu. SOC stock was calculated using the following formula:

(6) $$\mathrm{SOC}\;\text{Stock}={\mathrm{BD}}^{*}\;D^{*}\;\mathrm{OC}$$

where, SOC stock, BD, D, and OC represent soil organic carbon stock per unit area (ton ha^−1^), soil bulk density (g cm^−3^), the total depth at which the sample was taken (30 cm) and organic C concentration (%) respectively.

Bulk density (BD) was calculated using the following equation (Pearson 2007):

(7) BD=MS/VC

where BD, MS, and VC represent Bulk density (g cm^−3^), Mass of the dried soil (g), and volume of core sampler (cm^3^) respectively.

#### 2.4.3 Total Carbon Stock

The total C stock density was calculated by adding the C stock densities of the individual C pools using (Pearson 2007) formula as follows:

$$\mathrm{TC}\;\left(\mathrm{ton}\;{\mathrm{ha}}^{-1}\right)=\mathrm{AGC}+\mathrm{BGC}+\mathrm{SOC}$$

where, TC = Total C stock density for all pools (ton ha^−1^), AGC = C in above‐ground tree biomass (ton ha^−1^), BGC = C in below‐ground biomass (ton ha^−1^) and SOC = Soil organic C (ton ha^−1^).

### 2.5 Statistical Analysis

Microsoft Excel 2010 and R studio version 4.2.3 were used for all statistical analyses of the data. To ensure the quality and reliability of the dataset, outlier removal was conducted using the interquartile range (IQR) method. The IQR is calculated as the difference between the third quartile (Q3) and the first quartile (Q1), representing the middle 50% of the data. Observations were identified as outliers if they fell below the lower bound (Q1–1.5 × IQR) or above the upper bound (Q3 + 1.5 × IQR). For each variable of interest, Q1 and Q3 were computed, and any data points lying outside the defined boundaries were removed. This approach ensured that extreme values, which could disproportionately influence the results, were excluded while retaining the integrity of the dataset. All analyses were conducted on the cleaned dataset following the outlier removal process. Before statistical analysis, data were tested for normality (*p* > 0.05) and data were found to be normally distributed. C stock was considered a dependent variable, whereas topographical factors such as slope, aspect, and physiographic region were taken as independent variables. To evaluate the statistically significant differences (*p* < 0.05) in C stocks among C pools under each topographic factor, one‐way ANOVA was employed. To find significant pairs among the various independent variable categories, a post‐hoc Tukey test was used. Furthermore, all results are presented as mean ± SE to represent the variability within each category. The dataset used in the study is available in the Figshare repository (GC et al. 2025).

## 3 Results

### 3.1 Carbon Stocks Across Physiographic (Altitudinal) Regions

Mountain and High Himal (174.04 ± 29.75 ton ha^−1^) had the highest mean AGC, followed by Terai (124.59 ± 9.92 ton ha^−1^), Siwalik (111.39 ± 8.33 ton ha^−1^), and Mid Hill (102.38 ± 12.11 ton ha^−1^). Similar to AGC, Mountain and High Himal (34.04 ± 5.95 ton ha^−1^) had the highest mean BGC, followed by Terai (24.91 ± 1.98 ton ha^−1^), Siwalik (22.27 ± 1.66 ton ha^−1^), and Mid Hill (20.47 ± 2.42 ton ha^−1^). One‐way ANOVA revealed that AGC and BGC stocks in various physiographic regions showed significant differences (*p* < 0.05). Further, the post‐hoc test revealed that only the pairs Siwalik–Mountain and High Himal and Mid hill‐Mountain and High Himal were statistically different for AGC and BGC.

**FIGURE 3:**
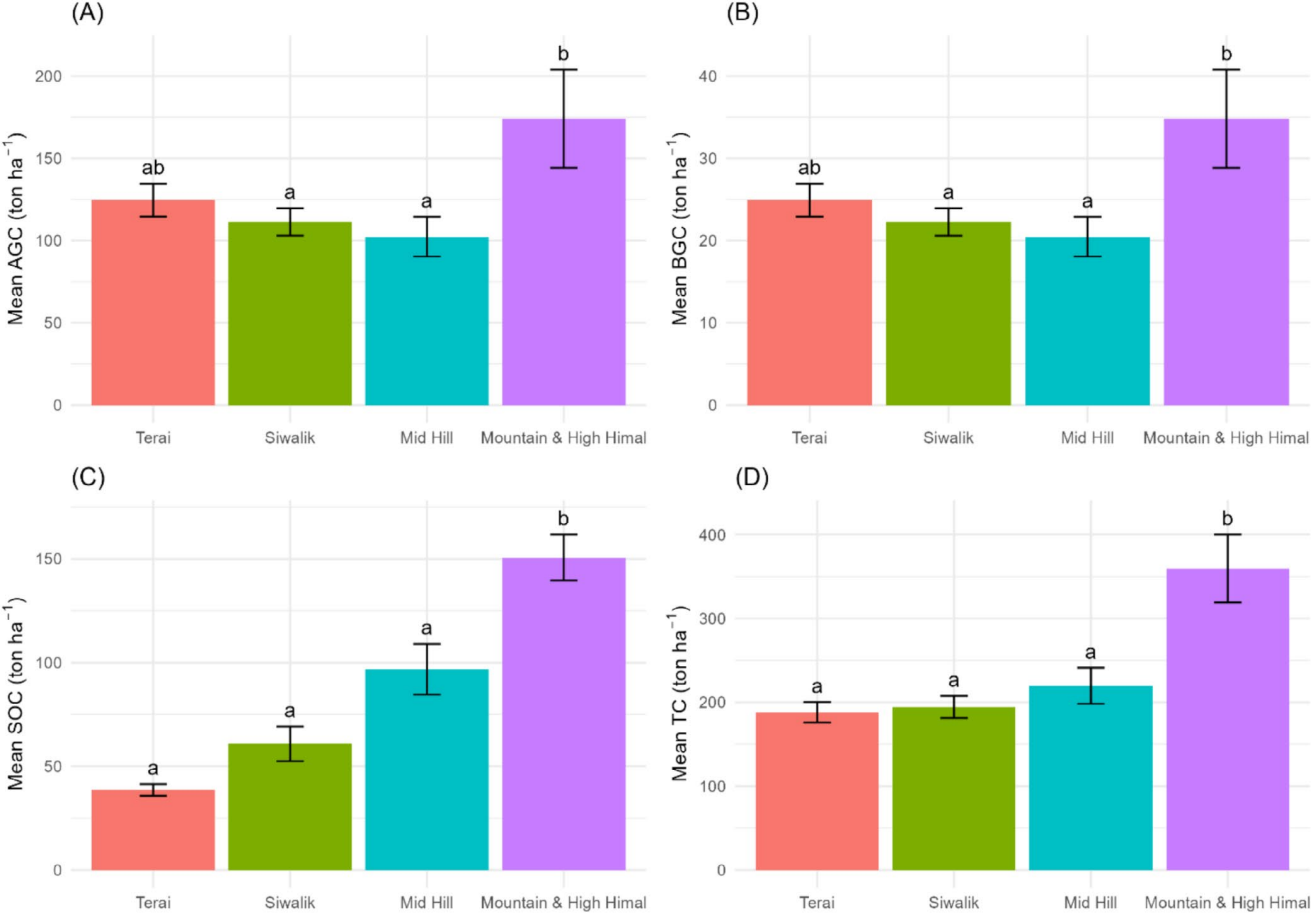
Mean soil organic carbon stock in different physiographic regions: Teri, Siwalik, Mid hill, and Mountain and High himal. Different letters indicate significant differences at 95% confidence intervals. Bars represent mean values, with error bars indicating ±SE.

On the other hand, SOC and TC stocks showed an increasing trend along the lower to higher physiographic regions (Figure 3). The SOC in Terai (38.61 ± 2.75 ton ha^−1^) was found to be the lowest, while Mountain and High Himal (150.62 ± 11.02 ton ha^−1^) had the highest SOC values (Figure 3). Similarly, TC was found to be highest in Mountain and High Himal (359.47 ± 40.44 ton ha^−1^), followed by Mid Hill (219.65 ± 21.45 ton ha^−1^), Siwalik (194.50 ± 13.20 ton ha^−1^) and lowest in the Terai region (188.12 ± 12.32 ton ha^−1^). One‐way ANOVA revealed significant differences in SOC and TC stocks across various physiographic regions (*p* < 0.05). Post‐hoc tests revealed that Mountain and High Himal had significantly higher SOC and TC compared to Siwalik, Mid Hill, and Terai. However, there was no significant difference (*p* > 0.05) in SOC among Terai, Siwalik, and Mid hill (Figure 3).

### 3.2 Carbon Stocks Across Different Aspect Categories

**FIGURE 4:**
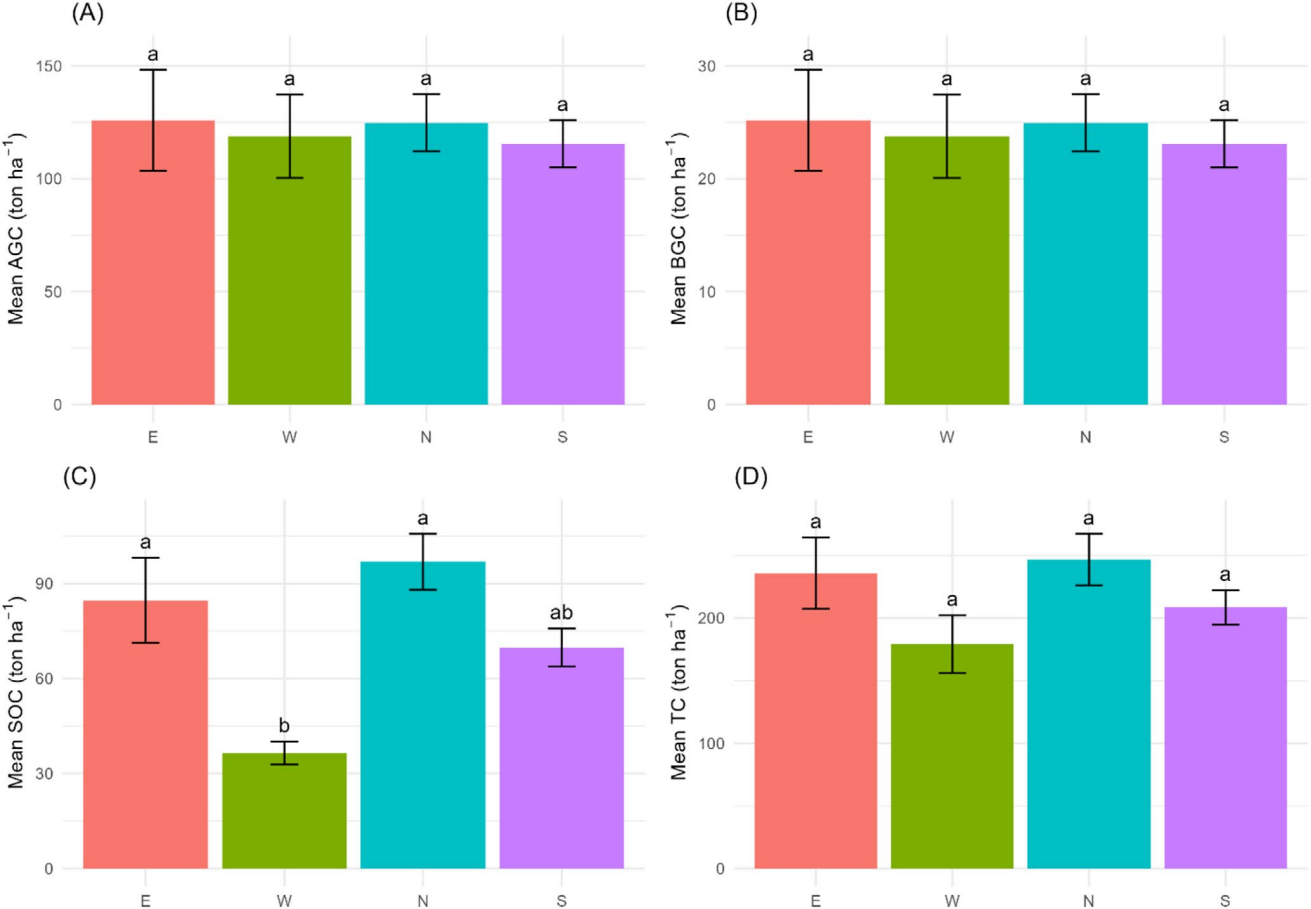
(A and B) Mean aboveground carbon stock in different aspects: East, West, North, and South. (C and D) Mean soil organic carbon stock in different aspects: East, West, North, and South. Different letters indicate significant differences at 95% confidence intervals. Bars represent mean values, with error bars indicating ±SE.

Our study found higher C stocks in E and N aspects than S and W aspects across all C pools (Figure 4). In terms of AGC, the E aspect exhibited the highest mean value (125.9 ± 22.34 ton ha^−1^), followed closely by the N aspect (124.82 ± 12.69 ton ha^−1^), while the S and W aspects had slightly lower values (115.51 ± 10.44 ton ha^−1^ and 118.87 ± 18.46 ton ha^−1^, respectively). The BGC showed similar trends across aspects, with minimal variation in mean values ranging from 23.1 ± 2.09 to 25.18 ± 4.47 ton ha^−1^. However, SOC displayed more noticeable differences, with the N aspect having the highest mean value (96.85 ± 8.82 ton ha^−1^), followed by the E aspect (84.73 ± 13.44 ton ha^−1^), the S aspect (69.82 ± 5.97 ton ha^−1^), and the W aspect (36.45 ± 3.63 ton ha^−1^). Finally, TC content varied across aspects, with the N and E aspects exhibiting the highest mean values (246.63 ± 20.5 and 235.81 ± 28.53 ton ha^−1^, respectively), while the S and W aspects had slightly lower mean values (208.44 ± 13.83 and 179.1 ± 23.04 ton ha^−1^, respectively). Additionally, there were significant differences in SOC levels among the aspects (*p* < 0.05), while AGC, BGC, and TC levels did not show significant variation (Figure 4). The post‐hoc Tukey test revealed that the differences in pairs W–E, S–N, and W–N were statistically significant, whereas the pairs N–E, S–E, and W–S had not statistically significant differences.

### 3.3 Carbon Stocks Across Different Slope Categories

**FIGURE 5:**
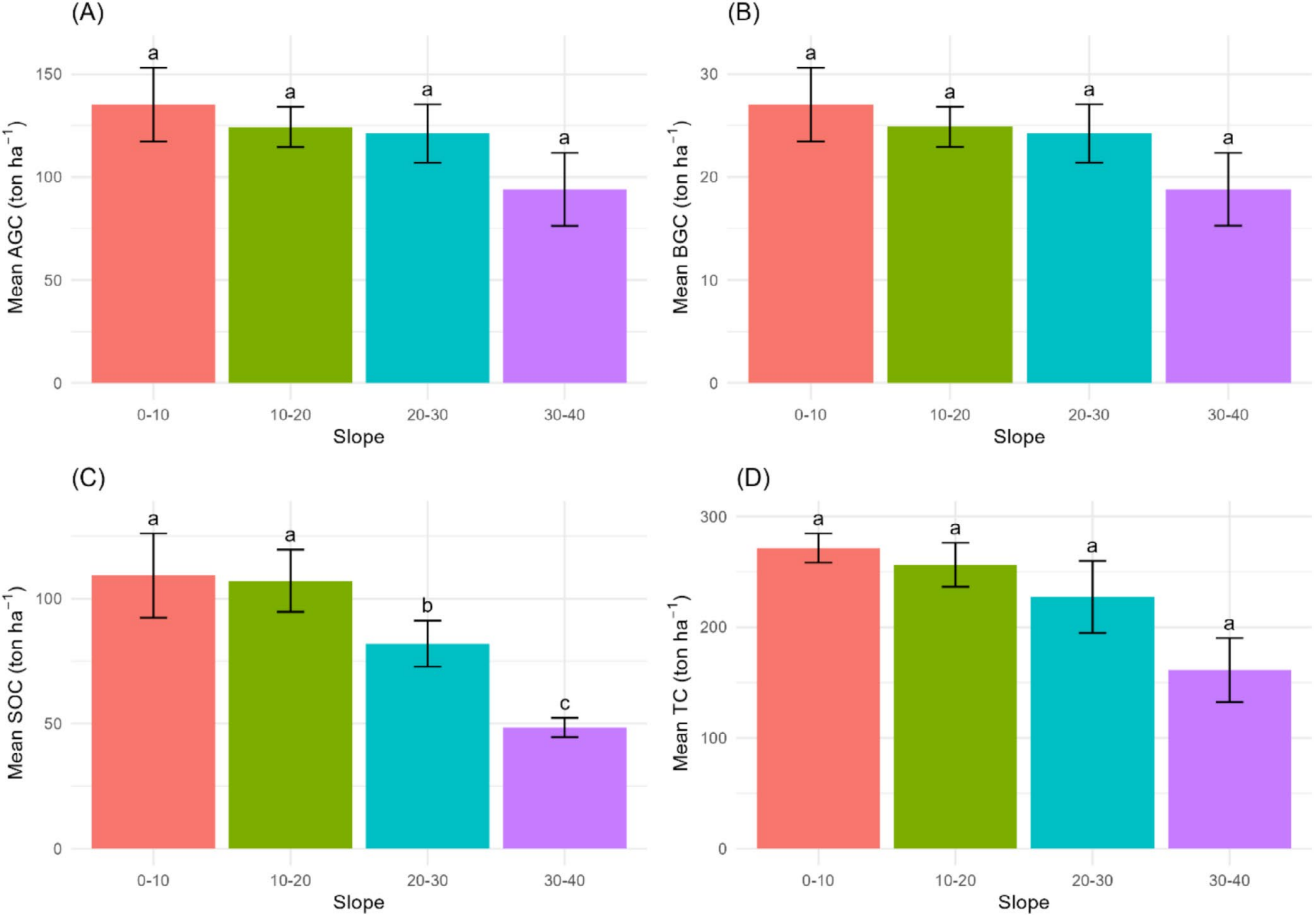
(A and B) Mean above‐ground carbon stock in different slopes: 0°–10°, 10°–20°, 20°–30°, and 30°–40°. (C and D) Mean soil organic carbon stock in different slopes: 0°–10°, 10°–20°, 20°–30°, and 30°–40°. Different letters indicate significant differences at 95% confidence intervals. Bars represent mean values, with error bars indicating ±SE.

We found a general trend of decreasing C content with steeper slopes across all C pools (Figure 5). Our study found the highest AGC at (0–10)° slope (135.17 ± 17.87 ton ha^−1^) while the lowest at (30–40)° slope (94.05 ± 17.69 ton ha^−1^). BGC was also found to be highest at (0–10)° slope (27.03 ± 3.57 ton ha^−1^) and lowest at (30–40)° slope (18.81 ± 3.53 ton ha^−1^). Similarly, SOC was found to be highest at (0–10)° slope (107.14 ± 12.51 ton ha^−1^) and lowest at (30–40)° slope (48.42 ± 3.84 ton ha^−1^). Lastly, the highest TC was found at (0–10)° slope (271.45 ± 32.58 ton ha^−1^) and the lowest at (30–40)° slope (151.23 ± 28.80 ton ha^−1^) (Figure 5).

One‐way ANOVA revealed a statistically significant difference in SOC stock across slope positions (*p* < 0.05), AGC and BGC showed no statistically significant differences (*p* > 0.05) (Figure 5). Post‐hoc Tukey test revealed that the pair (20–30)° slope and (0–10)° slope, as well as (20–30)° slope and (10–20)° slope, showed a significant difference (*p* < 0.05). Additionally, all other slope pairs showed significant differences compared to the (30–40)° slope (*p* < 0.05) (Figure 5).

## 4 Discussion

### 4.1 Mountain and High Himal Regions Store Higher Carbon Stocks

Aligning with our hypothesis, this study found the highest AGC and BGC stock in the Mountain and High Himal regions (Figure 3) followed by which aligns with previous studies (Thokchom and Yadava 2017; Nepal et al. 2023) (Figure 3). Higher AGC and BGC stock in the Mountain and High Himal could be due to the dominance of old, mature, large, and taller trees, despite the presence of lower species richness (Dani and Baniya 2020; Nepal et al. 2023). Similarly, lower AGC and BGC stock reported in the Mid‐Hill region could be due to the heavy reliance of local communities on forests for livelihoods, fuelwood, timber, and other essential resources in the Mid‐Hill regions (Tripathi et al. 2020; KC et al. 2021; Cedamon et al. 2022). This can result in higher rates of deforestation, land conversion, and degradation, ultimately reducing C stocks. Another reason for higher AGC and BGC stock in higher altitudes could be the lower levels of disturbance caused by both humans and animals in upper altitudes compared to middle and lower altitudes (Bhusal and Bhattarai 2020). In upper altitudes, the harsher environmental conditions, rugged terrain, and remoteness often limit human access and activities such as logging, agriculture, and infrastructure development (Singh and Verma 2018). Similarly, the density of grazing animals tends to decrease with increasing elevation due to factors like harsh climate, limited forage availability, and difficulty in accessing high‐altitude areas (Girma et al. 2014; Limbu and Koirala 2011). Similarly, this study found an increasing trend of SOC stock with increasing altitude (Figure 3). This spatial distribution pattern of SOC stock aligns with previous studies (Dorji et al. 2014; Tashi et al. 2016; Khanal et al. 2023). This might be explained by lower mean annual air temperature‐induced slower rates of decomposition, which lead to a greater long‐term build‐up of organic matter (leaf litter, wood debris, twigs) in the cold climatic zones of mountain forests than in the tropical lowlands (Khanal et al. 2023). Comparably, the lower altitude (Terai region) may have a reduced SOC content due to increased disturbance levels, livestock grazing, altered plant cover, the eradication of woody species, and the predominance of invasive species (Tolessa and Senbeta 2018). Higher total C stock in the Mountain and High Himal regions is due to its higher AGC, BGC, and SOC stock (Figure 3).

### 4.2 Carbon Stocks Across Different Aspect Categories

Our study reported higher AGC and BGC in N and E aspects than in W and S aspects (Figure 4) which aligns with previous studies (Simegn and Soromessa 2015; Teshager et al. 2018; Gashu 2022). Simegn and Soromessa (2015) discovered the lowest AGC and BGC in the S aspect (257.37 and 51.4738 ton ha^−1^) and the highest in the N aspect (296.98 and 59.39 ton ha^−1^), respectively, in the lowland region of Simien Mountains National Park, Ethiopia. Similarly, Teshager et al. (2018) reported higher values of both AGC and BGC in the N aspect (183.00 and 49.41 ton ha^−1^, respectively) compared to the W aspect (9.58 and 2.59 ton ha^−1^) of Weiramba Forest in the Amhara Region, Ethiopia. Furthermore, Gashu (2022) reported the largest AGC and BGC in the E (294.5 ± 15.5 and 58.9 ± 3.1 ton ha^−1^, respectively) and W aspect (255c ± 18 and 51 ± 3.6 ton ha^−1^, respectively) of Wacho Forest in the Hawa Galan district in southwestern Ethiopia. This might be because N and E aspects typically experience cooler temperatures compared to the warmer W and S aspects (Heithecker and Halpern 2007). Cooler temperatures in the N and E aspects may foster conditions more conducive to vegetation growth and biomass accumulation, thereby leading to higher aboveground carbon stock (Tsui et al. 2004; Sigua and Coleman 2010). Reduced solar radiation in these cooler regions helps retain moisture in the soil, supporting robust tree growth even during dry periods (Greiser et al. 2024). With sustained access to water and moderate temperatures, trees exhibit enhanced photosynthesis, resulting in increased production of biomass over time (Gil et al. 2012; Jin et al. 2011).

Consistent with the AGC and BGC, SOC stock also followed a similar trend (Figure 4), with higher carbon levels in the N and E aspects, which is in line with prior studies (Yohannes et al. 2015; Bangroo et al. 2017; Sharma et al. 2011). Yohannes et al. (2015) reported higher SOC in the E aspect (240.66 ± 10.5 ton ha^−1^) than in the S aspect (176.2 ± 20.46 ton ha^−1^) in Gedo Forest, West Shewa Zone of Oromia National Regional State, Ethiopia. Similarly, Bangroo et al. (2017) reported the highest SOC stock values (160.9 and 111 ton ha^−1^) along the N and S aspects in the 1800–2200 masl zone, while the 2200–2500 masl zone had only 107.7 ton ha^−1^ and 94.4 ton ha^−1^, respectively, in the Himalayan Mawer Forest Range, India. Additionally, Sharma et al. (2011) reported lower SOC in the S than in the N aspect in seven forest types of the temperate region of Garhwal Himalaya, India. This pattern could be attributed to several factors. Firstly, the cooler temperatures in the North and East aspects slow down decomposition rates, allowing organic matter to accumulate in the soil over time (Yohannes et al. 2015). Additionally, reduced solar radiation in these regions helps retain moisture in the soil, creating conditions favorable for the preservation of organic carbon (Cao 2018). Moreover, the cooler climate may promote the growth of vegetation with extensive root systems, which contribute to the input of organic matter into the soil through litterfall and root turnover (Yohannes et al. 2015). As a result, the North and East aspects accumulate greater SOC due to the synergistic effects of reduced decomposition, enhanced moisture retention, and increased organic matter input. This explains how the microclimates associated with different aspects can influence carbon dynamics. Therefore, understanding how temperature gradients affect vegetation growth, decomposition rates, and soil moisture levels across aspects can inform decisions about where to prioritize conservation efforts or implement land management practices aimed at enhancing carbon sequestration (Yohannes et al. 2015; Salunkhe et al. 2018; Rodrigues et al. 2023).

### 4.3 Carbon Stocks Decrease With Increasing Slope

Our study found decreasing trend of AGC, BGC, SOC and TC with increasing trend of slope. This aligns with Yohannes et al. (2015) in Gedo Forest of Ethiopia where they reported decreasing of AGC from 324.79 ± 32.59 ton ha^−1^ to 187.62 ± 13, 175 ± 7.8 ton ha^−1^ and decline of SOC from 195.87 ± 12.8 ton ha^−1^ to 175 ± 7.8 ton ha^−1^ in lower and higher slope respectively. Further, our results confirmed the existing evidence in Marshall et al. (2012) and Olson (2010) conducted in tropical Tanzania forest and southern Illinois where AGC and SOC were higher in shallow slopes than the steep, respectively. This might be due to decrease in floral richness with increasing slope (Gubena and Sormessa 2017; Maggi et al. 2005). Similarly, steeper slopes may limit the ability of certain plant species to accumulate biomass, as they might face challenges in accessing sunlight, nutrients, and water (Sarker et al. 2015). This limitation in biomass accumulation directly affects AGC stocks. Additionally, less vegetation coupled with decrease in soil infiltration promotes soil erosion due to accelerated runoff (Yohannes et al. 2015; Begum et al. 2014). As wind and water erosion can transport soil particles, including organic matter, away from the site, which might result in lower soil C (Lal 2019). However, some studies have also indicated a positive correlation (Ajami et al. 2016; Hou et al. 2020), suggesting that, in certain contexts, C stock may increase with slope gradient. In addition, Dar and Parthasarathy (2022) found no significant correlation between slope and tree C. These divergent findings highlight the complexity of the relationship between slope gradient and C stock, and they may stem from variations in factors such as mean annual precipitation, mean annual temperature, management practices, disturbances, and site‐specific tree species (Boča et al. 2014; James and Harrison 2016; Kalies et al. 2016; Zhang et al. 2013). Therefore, site specific study is needed to infer conclusion about slope‐C relation and implementation of site‐specific management activities for C storage maximization.

### 4.4 Sources of Uncertainty and Its Management

Uncertainties in this study primarily stemmed from field measurements, the selection of allometric models, and the extrapolation of findings to larger scales. Measurement errors in tree dimensions such as tree height and DBH were mitigated by following standardized data collection protocols (DFRS 2014). To ensure robustness, validated tree‐specific allometric models were developed for Nepalese tree species (Sharma and Pukkala 1990) and the Walkley‐Black method was used for assessing biomass and SOC, respectively. The sampling design was optimized to address spatial variability, while statistical methods were employed to account for potential biases in extrapolation. A hybrid approach was adopted for the forest inventory, involving satellite image interpretation in the first phase and field measurements of forest characteristics in the second phase (DFRS 2014). A two‐phased sampling method was used to concentrate field measurements on forested clusters and minimize logistical challenges, such as accessing nonforested clusters (DFRS 2014). Reliability was quantified using the standard error of the mean stem volume, with the desired accuracy set at 10% at the 95% confidence level. Data supporting this assessment were collected from permanent sample plots established by the Department of Forest Research and Survey, Nepal. Additional details are available in the report published by the Department of Forest Research and Survey, Nepal (DFRS 2014).

Despite these measures, residual uncertainties persist due to unmeasured environmental variables (e.g., soil properties, microclimatic factors) that are difficult to measure or account for fully, despite efforts to control for key variables like aspect, slope, and elevation. One such factor is soil variability, where unmeasured differences in soil properties such as nutrient content, organic matter, and water retention can influence tree growth and C accumulation. For example, plots on lower slopes may have nutrient‐rich soils that support faster tree growth and higher C stocks compared to higher elevations with poorer soils. Additionally, microclimatic differences contribute to residual uncertainty, as variations in sunlight exposure, temperature, and humidity across aspects and slopes can affect tree productivity. South‐facing slopes, receiving more sunlight, may exhibit faster growth and higher carbon storage than shaded north‐facing slopes, while cooler temperatures at higher elevations may reduce biomass accumulation.

### 4.5 Implication of the Study Findings

Our study provides valuable insights into the spatial variations of C stock and its relationship to land attributes in the far‐western region of Nepal. These findings can guide the identification of high C stock areas and regions with significant soil C accumulation. This can assist managers in prescribing effective C conservation activities for community user groups as well as private forest holders. The far‐western region of Nepal covers approximately 16.94% (1,010,207 ha) of the total Forest of Nepal, which was categorized in slope, aspect, and physiographic categories for organic carbon storage assessment in this study. Therefore, incorporating the results of this study into regional and national C accounting systems will significantly reduce uncertainties in biomass and soil C estimates. For example, prior to this study, carbon stock assessments in the far‐western region of Nepal were primarily conducted at the community forest level or within small, localized areas (Pandey et al. 2019; Joshi et al. 2020, 2021). Our findings, which provide species‐specific insights and account for spatial variations in C stock related to land attributes, can refine these estimates. These insights can help refine national inventories under frameworks like reducing emissions from deforestation and forest degradation (REDD+), supporting more accurate monitoring, reporting, and verification (MRV) processes. For instance, using the study's findings, forest managers can better target areas with higher C sequestration potential for conservation efforts, improving C credit calculations and ensuring more accurate monitoring. By enhancing the credibility of national C accounting, this research also contributes to more reliable reporting to the UNFCCC (United Nations Framework Convention on Climate Change), where countries submit their greenhouse gas inventories. This improves the transparency and effectiveness of climate change mitigation efforts, such as forest C offset projects under REDD+, by ensuring that C credits issued are based on precise, up‐to‐date data.

## 5 Conclusions

This article reports the quantification of C variation along selected topographic factors in Far‐western Nepal. A notable spatial heterogeneity in C stocks, with the Mountain and High Himal regions exhibiting the highest mean AGC, BGC, as well as SOC stocks, is explored. Furthermore, aspect emerged as a significant determinant, with the East aspect demonstrating the highest AGC and BGC stocks, while the North aspect exhibited the highest SOC stock. Moreover, slope is identified as a crucial factor affecting C stocks, with declines observed in C stocks as slopes steepened. Particularly, the (0–10)° slope category displayed the highest C stocks across all pools, whereas the (30–40)° slope category exhibited the lowest C stocks. Given the influence of these topographical factors on C stock necessitates the implementation of site‐specific forest management strategies that account for the unique topographical characteristics of each area ultimately contributes to the sustainable management of forest ecosystems in Far‐western Nepal and beyond. This study examined SOC stock up to 30 cm and included limited C pools. Therefore, future research needs to explore soil depth to 1 m and include all C pools for a comprehensive understanding of forest C stock. Additionally, longitudinal studies are recommended to further understand the interplay between topographical factors and C distribution over time.

## Conflicts of Interest

The authors declare no conflicts of interest.

## Data Availability

The datasets generated and analyzed during the current study are available in the Figshare repository (GC et al. 2025) at https://doi.org/10.6084/m9.figshare.28188713.
